# Supplementary figures and images for: Induction of long-lived potential aestivation states in laboratory An. gambiae mosquitoes
Source: Parasit Vectors. 2020 Aug 12;13:412. doi: 10.1186/s13071-020-04276-y (PMC7424682; doi:10.1186/s13071-020-04276-y)

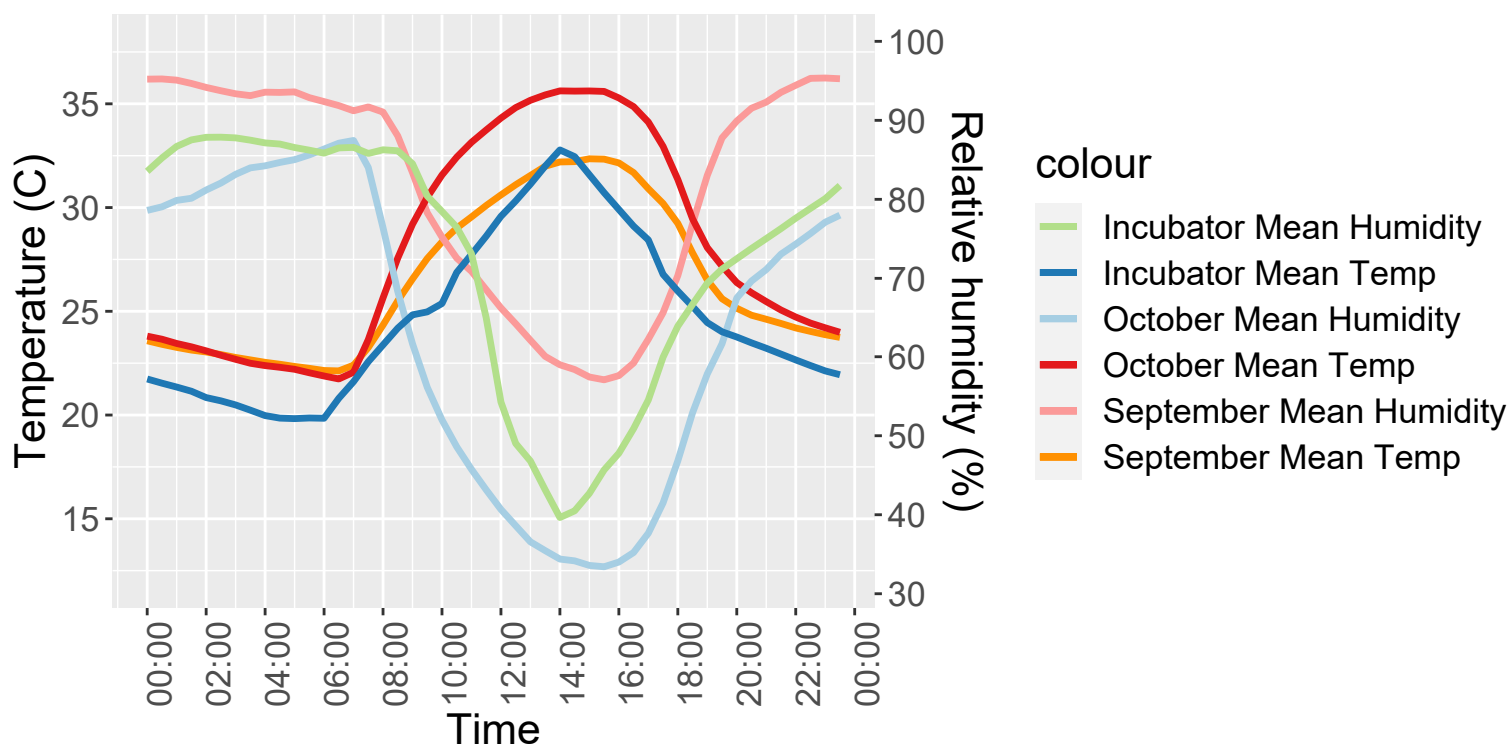

Supplement: Supplementary file 1 — Additional file 1: Figure S1. Temperature and Humidity profiles for Thierola, Mali (red, orange) and the priming incubator (green, blue) as recorded by a HOBO temperature and humidity logger. [file 13071_2020_4276_MOESM1_ESM.pdf]

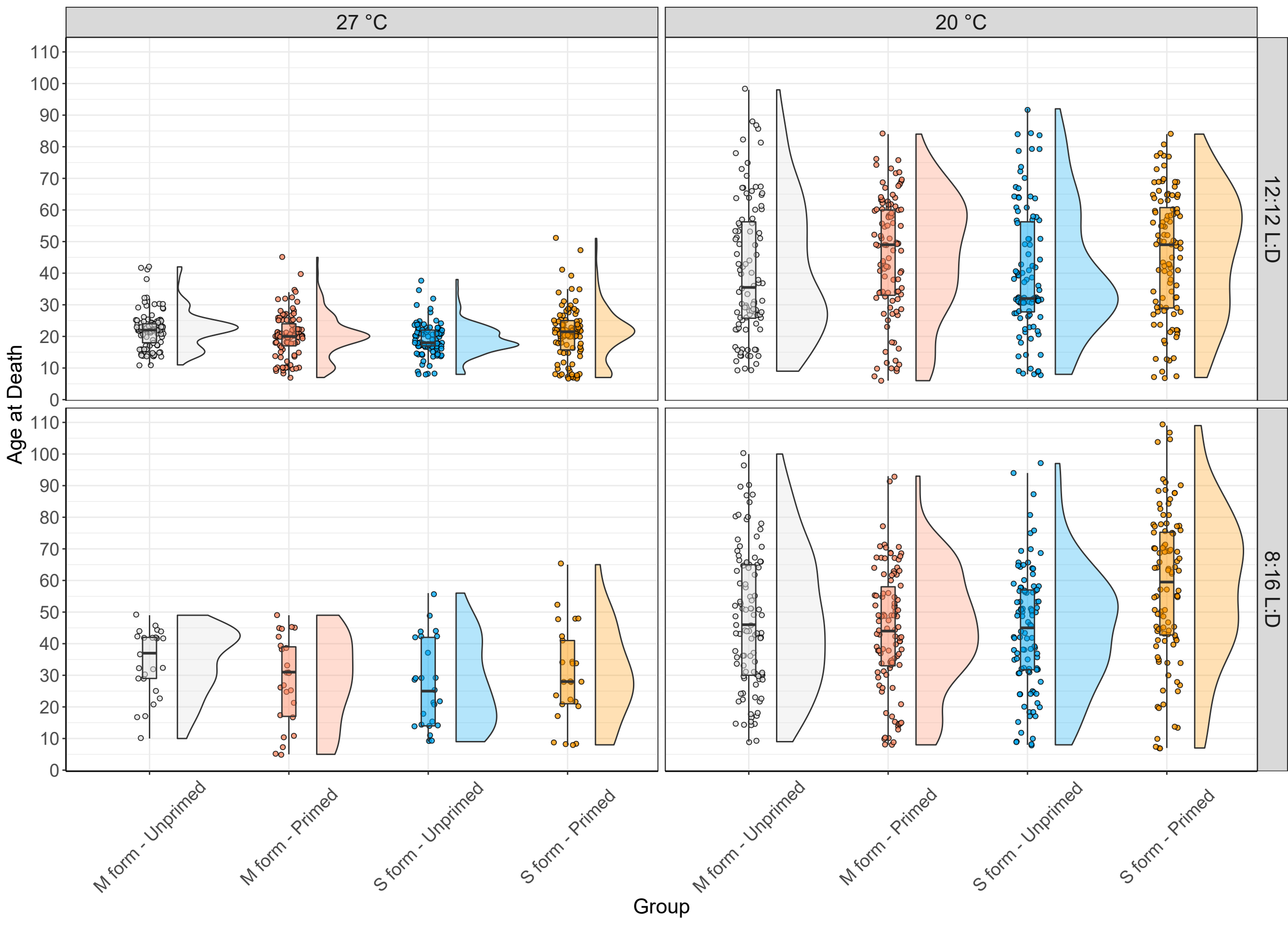

Supplement: Supplementary file 2 — Additional file 2: Figure S2. Boxplot and raincloud [61] plots showing the date of death ranges and distributions for each of the experimental groups. [file 13071_2020_4276_MOESM2_ESM.pdf]

a

Wing Area Round 2

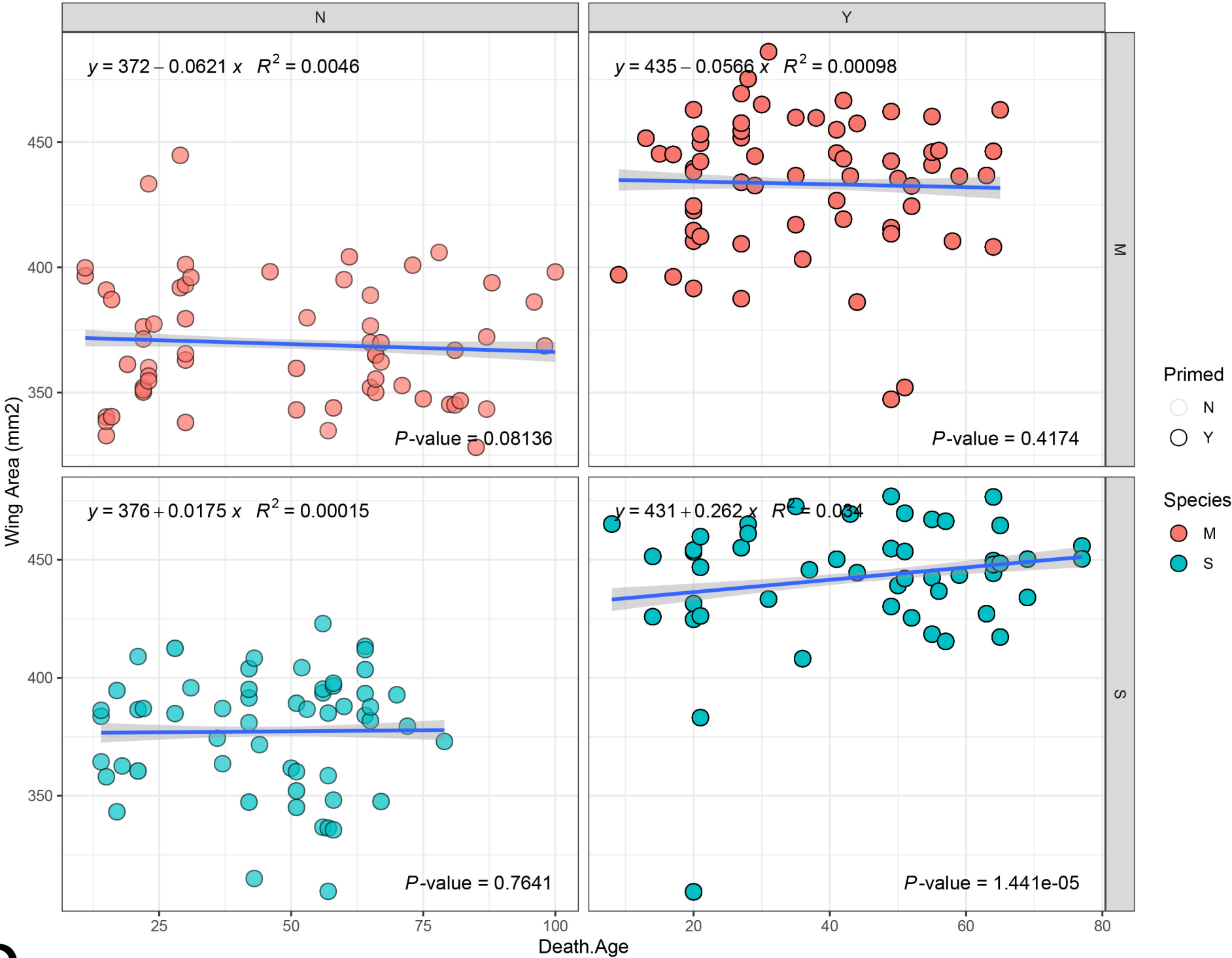

b

Wing Area Round 3

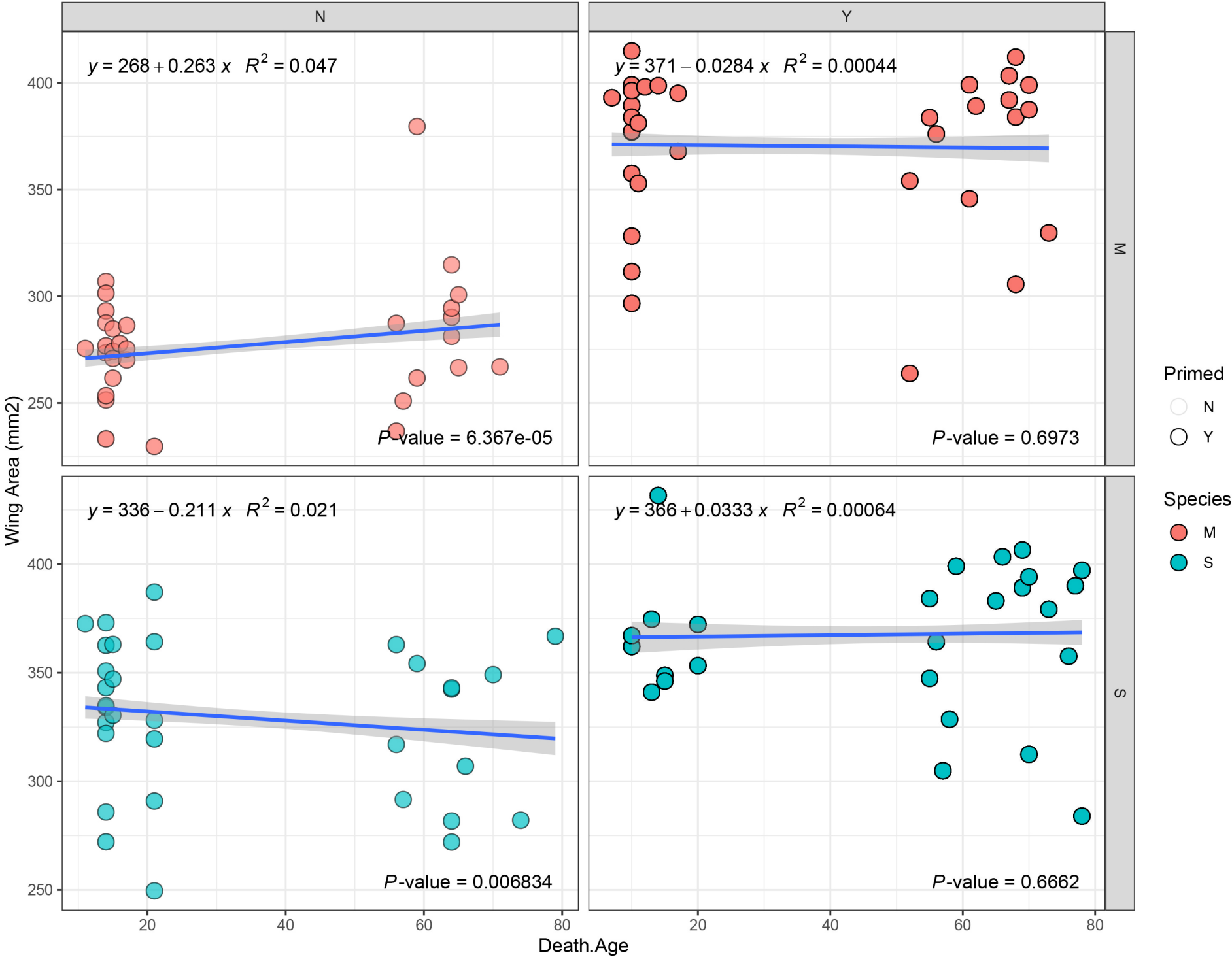

Supplement: Supplementary file 5 — Additional file 5: Figure S4. Correlations of wing areas and survival for replicates 2 and 3 of the experiment. No consistent correlations between wing area and lifespan were found between the two experiments. [file 13071_2020_4276_MOESM5_ESM.pdf]
