# Supplementary material for: Induction of long-lived potential aestivation states in laboratory An. gambiae mosquitoes
Source: Parasit Vectors. 2020 Aug 12;13:412. doi: 10.1186/s13071-020-04276-y (PMC7424682; doi:10.1186/s13071-020-04276-y)
Supplement: Supplementary file 3 — Additional file 3: Table S1. Akaike’s information criterion values for distributions tested using accelerated failure time models. [file 13071_2020_4276_MOESM3_ESM.docx]

**Additional file 3: Table S1.** Akaike’s Information Criterion values for distributions tested using accelerated failure time models.

| **distribution** | **chi.sq** | **df** | **AIC** | **dAIC** |
| --- | --- | --- | --- | --- |
| **weibull** | **721.325** | **9** | **9999.907** | **0** |
| loglogistic | 644.2013 | 9 | 10111.28 | 111.3704 |
| loggaussian | 526.0453 | 9 | 10151.48 | 151.5753 |
| lognormal | 526.0453 | 9 | 10151.48 | 151.5753 |
| rayleigh | 563.5402 | 8 | 10156.24 | 156.3284 |
| logistic | 585.1468 | 9 | 10410.19 | 410.2807 |
| gaussian | 528.9348 | 9 | 10418.64 | 418.7292 |
| t | 607.9373 | 9 | 10433.22 | 433.3162 |
| extreme | 717.2935 | 9 | 10634.63 | 634.7221 |
| exponential | 143.5639 | 8 | 11271.9 | 1271.996 |
