# Supplementary material for: Induction of long-lived potential aestivation states in laboratory An. gambiae mosquitoes
Source: Parasit Vectors. 2020 Aug 12;13:412. doi: 10.1186/s13071-020-04276-y (PMC7424682; doi:10.1186/s13071-020-04276-y)
Supplement: Supplementary file 4 — Additional file 4: Figure S3. Raincloud plot indicating measured wing lengths as a proxy for body size for primed and unprimed mosquitoes. Boxplot, individual points, and density distribution are shown. Point shapes indicate which replicate the lengths are from. [file 13071_2020_4276_MOESM4_ESM.pdf]

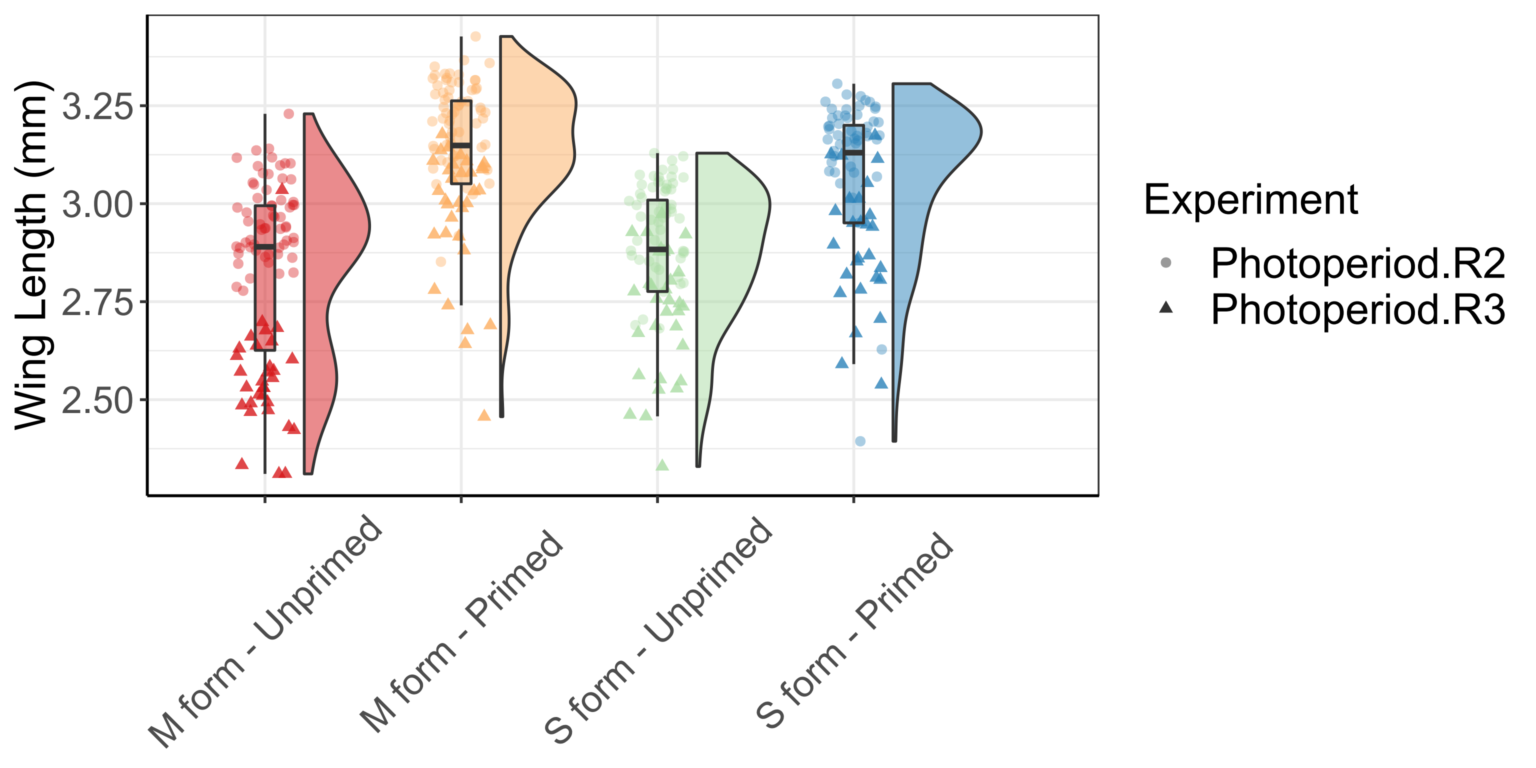

Wing Length (mm)
3.25
3.00
2.75
2.50
M form - Unprimed
M form - Primed
S form - Unprimed
S form - Primed
Experiment
Photoperiod.R2
Photoperiod.R3
